# Specific morphological aspects of the teeth and alveolar bone in Class II/2 malocclusion

**Published:** 2009

**Authors:** Radu Stanciu, Anca Temelcea, Ileana Simion, Dragos Stanciu

**Affiliations:** * ”Carol Davila” University of Medicine and Pharmacy – Faculty of Dentistry , Bucharest, Romania

**Keywords:** anterior deep bite, morphologic factors, genetic origin, Class II/2 malocclusion

## Abstract

In the genesis of dental-maxillary anomalies both morphologic factors (of proven genetic origin) and functional factors (as added ethio-pathogenic elements) are incriminated.

From this point of view, Class II/2 malocclusion, presents two morphologic factors whose genetic origin is certain and upon which functional factors have minor influence. These are: the morphology of the teeth and alveolar process in the anterior region of the maxilla and the type of rotation. The combination of the two generates a broad range of deep bite case variants.

The morphology of teeth and alveolar process in the anterior region of the maxilla and the type of rotation are the factors that, when influenced by individual specific growth and adaptation mechanisms, combined, generate a broad range of deep bite case variants. 

The first factor, the morphology of the teeth and alveolar process in the anterior region, presents characteristic aspects that make the malocclusion easy to point out among the dento – maxillary anomalies.

The modifications refer to the aspect of the crown of the frontal teeth, which have a narrowed labio-bucal diameter, with a concave labial face, without cingulum, creating the aspect of a crown in the shape of a shovel (Chateau).

We must notice that the crown axis forms an angle of 10-15° with the root axis, which gives the aspect of upper frontal retro-clination, term which should actually refer only to the crown aspect and its slope on the occlusion plane (**Fig. 1**).

**Fig. 1:**
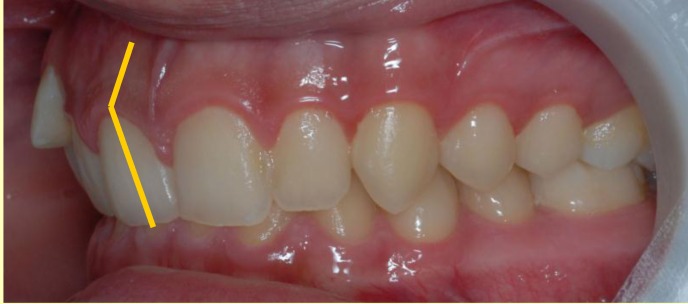
Crown axis forms with the root axis an angle of 10-15°, which gives the aspect of upper frontal retro-clination

The roots of the teeth in the upper front are usually strong, this makes them resist well to the occlusal forces (vertical forces) on the labial slope , producing, in the absence of the occlusion stop (represented by cingulum), very strong horizontal forces.

The typical angulation, as well as the absence of the occlusion stop, determines very important modifications, both in the upper alveolar bone, and at inter-maxillary relation level, in sagittal and vertical plane, modifications which might generate special functional disturbances.

A pro-alveolia specific to this type of malocclusion can be noted in the upper dental-alveolar region, with the accentuated highlighting of the position of the frontal teeth’s roots, in the form of labial hyper-calcification (**Fig. 2**). The aspect is typical and probably generated, in its turn, by the horizontal forces determined by the close occlusal contact between the incisors.

**Fig. 2:**
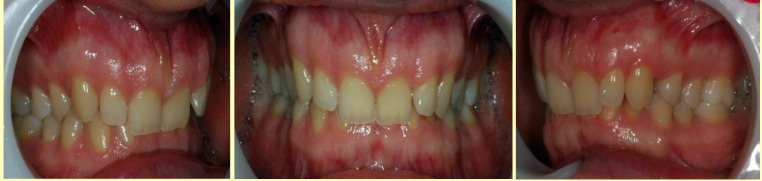
Pro-alveolia specific to this type of malocclusion, with the accentuated highlighting of the position of the frontal teeth’s roots, in the form of labial hyper-calcification

Upper incisor position is also typical and has two variants: the two central’s retroclined, and the two laterals are labially tilted and rotated (**Fig. 3**). The second form is represented by the retracted position of the four incisors and labial position of the two canines (**Fig. 4**). Of course, the retracted position in the frontal area generates, almost always, a lack of space, which leads to rotations of the teeth in the frontal group.

**Fig. 3:**
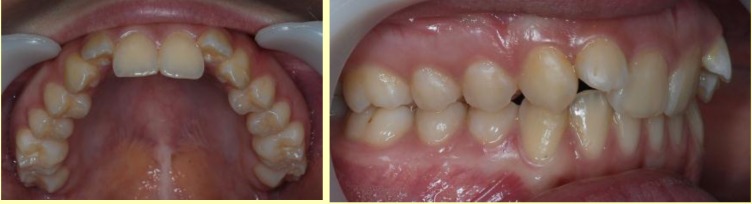
The two central’s retroclined, and the two laterals are labially tilted and rotated

**Fig. 4:**
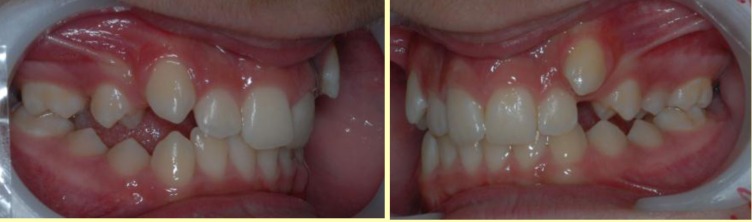
The retracted position of the four incisors and labial position of the two canines

Another characteristic element of special morphological importance is the form and position of the upper alveolar process and of the upper dental arch in the lateral areas. Thus, in the hypo-divergent pattern, there is important exo-alveolia, with wide alveolar processes, and the dental arches are tilted inwards (**Fig. 5**). In the other extreme, in the hyper-divergent variant, there is important endo-alveolia, with the crows of the upper lateral teeth tilted buccaly.

On the other hand, the absence of the cingulum in the upper incisors (no occlusal stops) determines, in vertical plan, a deep bite with the upper incisors covering the whole length of the crown of the already much extruded lower incisors (**Fig. 6**).

The contact of the frontal teeth generates, at the level of the lower jaw, three typical aspects, such as: mandibular retraction, with a normal alveolar development and, possibly, a slight frontal retroclination of the teeth; a normal sagittal position of the mandible, having very important modifications in the lower dental-alveolar process, which is an important retro-alveolar position, resulting in an anterior flattened dental arch (trapeze – **Fig. 7**); a mandibular normal position with retro-alveolar position, the lower dental-alveolar arch slightly modified in shape positioned to the posterior, on the jaw base.

**Fig. 5:**
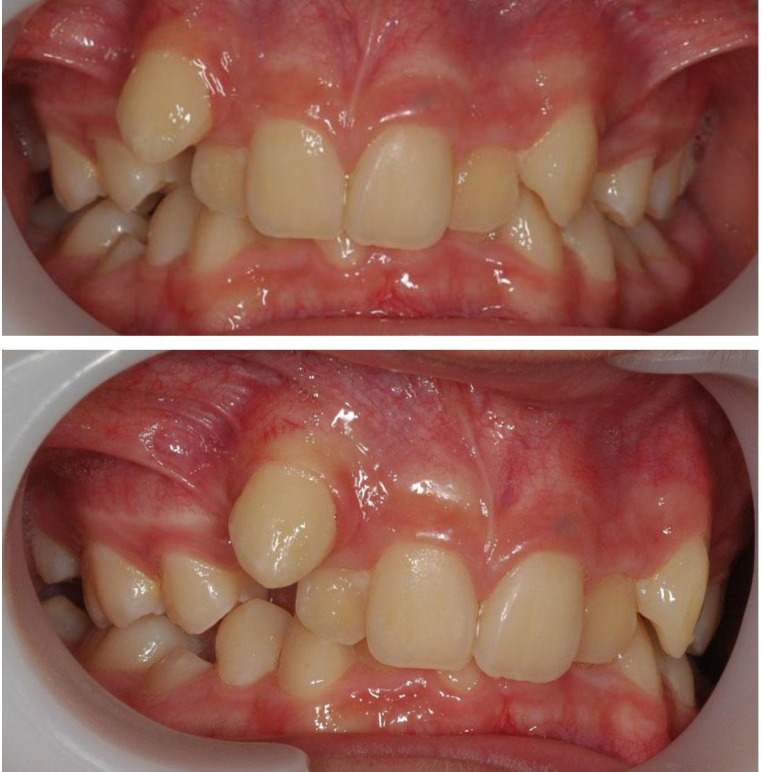
Important exo-alveolia, with wide alveolar processes, with the dental arches tilted inwards

**Fig. 6:**
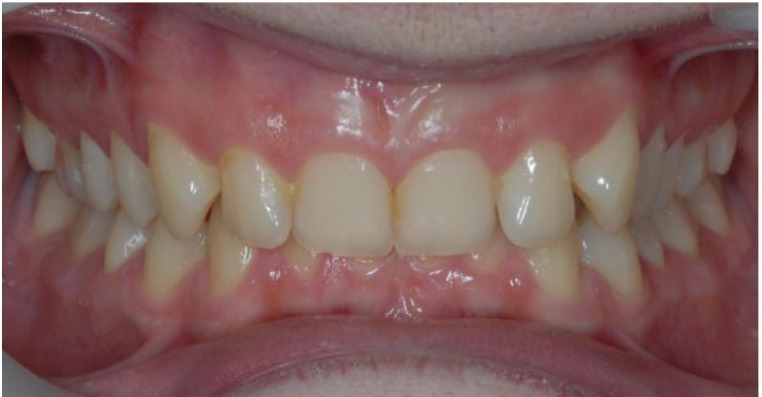
Deep bite with the upper incisors covering the whole length of the crown of the already much extruded lower incisors

**Fig. 7:**
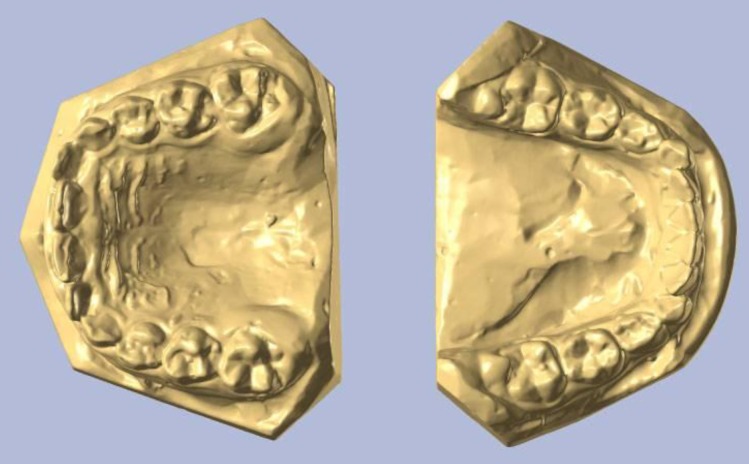
Flattened anterior dental arch - trapeze

Another very complex morphologic factor is represented by the facial and mandibular rotation type, which the parents passed on to the subject.

Thus, there are two types of Class II/2 malocclusion described, which, have the above-described dental and dental-alveolar characteristics, which take the shape of two distinct clinical entities.

The anterior rotation type (**Fig. 8**), in which the dimension of the lower anterior face is decreased, with a closed mandibular angle, with a horizontal occlusion plane and a mandibular plan, and the posterior rotation type (**Fig. 9**), in which the elements described above are reversed.

**Fig. 8:**
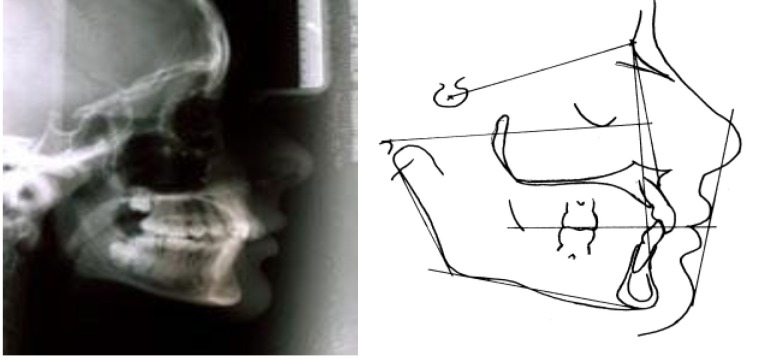
The anterior rotation type

**Fig. 9:**
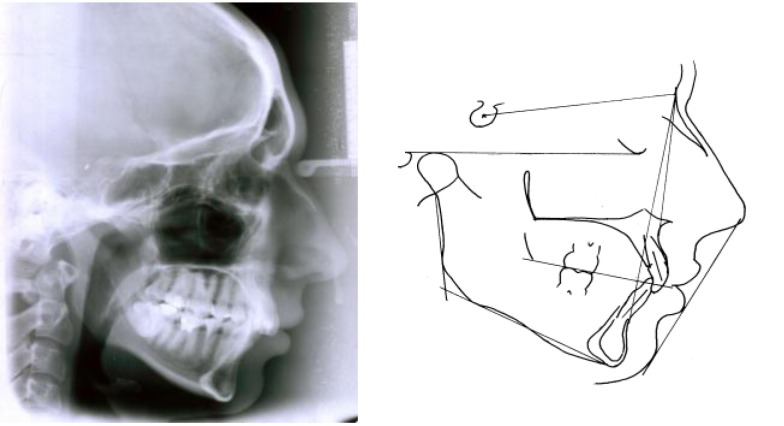
The posterior rotation type

Of course the elements described by Björk in the facial and mandibular rotation can balance or decompensate each other, but the frontal dental-alveolar aspects described above will be found as pathognomonic elements.

From the genetic point of view, the combination of these elements made some authors list more than 4300 forms of deep bite, all having as common characteristics the following three elements: “en-pelle” upper incisors, angulated crowns, hyper- frontal upper vestibular calcification.

In conclusion, each element taken separately can constitute, from the therapeutic point of view, another reason why the knowledge about the morphology of the dental-alveolar modifications is very important, even more since the early treatment of the anterior deep bite can create the conditions for resuming a normal growth pattern.
